# Evolutionary Wavelet Neural Network ensembles for breast cancer and Parkinson’s disease prediction

**DOI:** 10.1371/journal.pone.0192192

**Published:** 2018-02-08

**Authors:** Maryam Mahsal Khan, Alexandre Mendes, Stephan K. Chalup

**Affiliations:** Interdisciplinary Machine Learning Research Group (IMLRG), School of Electrical Engineering and Computing, The University of Newcastle, Callaghan, NSW 2308, Australia; Maharshi Dayanand University, INDIA

## Abstract

Wavelet Neural Networks are a combination of neural networks and wavelets and have been mostly used in the area of time-series prediction and control. Recently, Evolutionary Wavelet Neural Networks have been employed to develop cancer prediction models. The present study proposes to use ensembles of Evolutionary Wavelet Neural Networks. The search for a high quality ensemble is directed by a fitness function that incorporates the accuracy of the classifiers both independently and as part of the ensemble itself. The ensemble approach is tested on three publicly available biomedical benchmark datasets, one on Breast Cancer and two on Parkinson’s disease, using a 10-fold cross-validation strategy. Our experimental results show that, for the first dataset, the performance was similar to previous studies reported in literature. On the second dataset, the Evolutionary Wavelet Neural Network ensembles performed better than all previous methods. The third dataset is relatively new and this study is the first to report benchmark results.

## Introduction

Breast cancer is the second leading cause of cancer-related deaths in Australian women [1], while Parkinson’s disease is the second most common neurological condition in Australia [2]. The identification and assessment process for both diseases is multi-staged, that is tedious, time-consuming, and challenging where data needs to be manually labeled. Such assessments might also lead to misdiagnosis. In medical practice, in order to reduce the risk of misdiagnosis, opinions from multiple doctors (or specialist doctors) are taken into account. A similar approach is used in the computational intelligence domain, where performance of prediction models (or specialist models) is improved by combining multiple models, thus creating an ensemble of classifiers [3].

Ensemble classifiers and their use have been an active area of research for the past two decades, with Bagging [3] and Boosting [4] being two popular techniques, particularly in the field of applied statistics, pattern recognition and machine learning [5–7]. Many of the prediction models have been improved by using ensembles of support vector machines [8, 9], latent class analysis (LCA) [10], artificial neural networks [11], k-nearest neighbour [12], and even combinations of these classifiers [6].

Wavelet Neural Networks (WNN) are complex machine learning algorithms that use wavelet analysis and neural networks to generate prediction and control models. WNNs have been applied before in several areas, including time-series prediction and control [13, 14]. Evolutionary Wavelet Neural Networks (EWNN) are a recently proposed method for training WNNs and have been used to generate models for breast cancer and Parkinson’s disease classification [15]. However, there have been no studies on the prediction performance of an ensemble of EWNN classifiers, yet.

The motivation of this research is to evaluate the performance of EWNN and ensembles of EWNNs (EWNN-e) and compare them with other ensemble techniques used on the same data reported in literature. The findings of this paper aim to provide future researchers an alternative and effective model to compare with. Moreover, this study also investigates a newly published Parkinson’s disease dataset with multiple speech recordings.

The paper is organized as follows. Background provides an overview to Wavelet Neural Networks and its structure, EWNN and its response to a two-spiral task, related work on pruning ensembles, and description of some of the performance measures used in our study. The biomedical datasets, proposed mechanism and the experimental setup are described in the Experimental Methodology section. Results & Discussion presents the outcome of experiments and compares the method’s effectiveness with other techniques reported in literature. That section is then followed by the conclusions and future work.

## Background

### Wavelet Neural Networks

**Fig 1:**
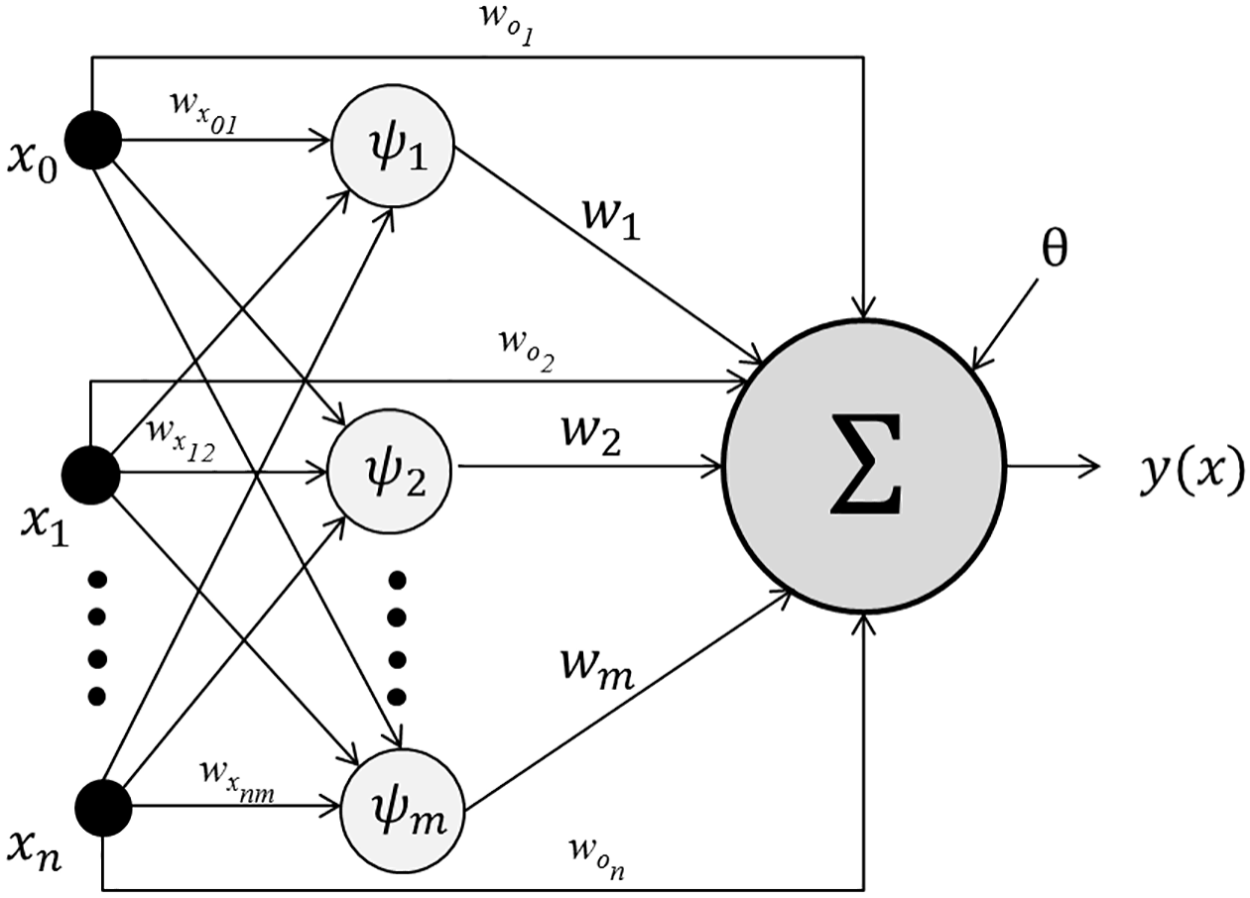
Structure of a Wavelet Neural Network. The network has *n* inputs in the input layer, *m* wavelons in the hidden layer and one neuron in the output layer. A bias *θ* is added to the WNN output response. Also, notice the *n* shortcut connections from the inputs to the output neuron.

Wavelet Neural Networks are a class of neural networks that combine the theory of wavelets and neural networks [16]. In standard neural networks, weights and biases are the only parameters that are trained and the most common activation functions used are sigmoid, hyperbolic tangent and linear functions. The activation functions found in WNNs are those that belong to the family of wavelet basis functions, with the most common being the *Morelet* and *Mexican hat*. In addition to weights and biases, three other parameters are used in WNNs: translate, dilate and rotate. The use of standard gradient methods to adjust WNN parameters, in particular the weights, biases, the translate and dilate parameters, often resulted in premature convergence [16, 17]. For that reason, global optimization approaches, such as genetic algorithms and evolutionary programming techniques, have been used in applications such as air and ground traffic flow [18, 19], energy consumption [20], large scale function estimation [21], function approximation [22] and power transformer monitoring [23]. A diagram of a WNN is shown in Fig 1. WNNs generally have a feed-forward structure, with one hidden layer having *m* wavelons (*ψ*_*m*_) and a neuron in the output layer. There are also *n* shortcut connections from the inputs to the output neuron.

### Evolutionary Wavelet Neural Networks

EWNNs were first proposed by Khan et al. [15] as a method for optimizing all WNN parameters concurrently. The method was tested successfully on both simulated and real datasets [15]. For a detailed description of EWNN characteristics and performance, we refer the reader to reference [15].

**Fig 2:**
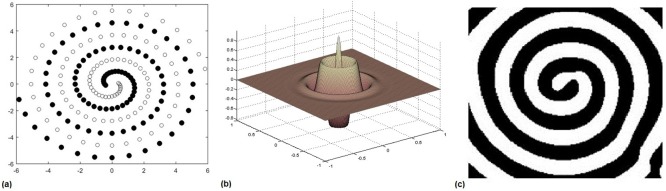
Training of an EWNN on a two-spiral task. (a) two-spiral classification task, each spiral consisting of 97 data points in the 2D Cartesian space. (b) Morelet wavelet activation function and (c) Optimal response on the task where a EWNN with Morelet wavelet activation function has separated the two classes successfully.

Fig 2 is an example of the EWNN applied to a standard benchmark two-spiral task shown in Fig 2(a). Two-spiral is a non-linear task with two spirals (shown as black and white dots) each with 97 sample data points in a 2D Cartesian space [24, 25]. The two-spiral task is fairly a challenging problem where for an Artificial Neural Network (ANN) with architecture 2-5-5-5-1 took 10,000–20,000 epochs in [24]. While in [26] a 2-50-1 ANN was trained by employing a second-order Newton optimization method where training took only 650 epochs. In contrast, for EWNNs with a wavelet activation function of Morelet shown in Fig 2(b), the optimum response of the EWNNs was achieved within 9 generations and with two wavelons only. This indicates its potential to separate non-linear classes effectively and efficiently.

### Classifier ensembles and pruning

The role of a classifier *C* is to learn how to map the feature set to a set of class label(s). The data samples are divided into training *U* and test *V* sets. The *C* is first trained on *U* where it learns the mapping process and then the performance of the *C* is measured on *V*. A multiple classifier system, or ensemble (*Ens*), is composed of a set of base classifiers that are trained on the same training dataset, and combined in a manner that improves the classification performance of the system. There are two main methods for creating an ensemble: averaging and voting [27]. Averaging is normally used for classifiers with numeric outputs. While voting is used for categorical outputs (e.g. binary), and is used in the present study. Each sample is classified independently by the *k* classifiers that constitute the ensemble. The final outcome of classification will be the most represented class labels. It is the one that received the most votes. The ensemble *Ens* classification for a sample *V* is described in Eq 1 (for the binary classification case).

$$\begin{array}{c} Ens\left(V\right)=\{\begin{array}{cc} 1, & \;\text{if}\;\sum_{i=1}^{k}C_{i}\left(V\right)>\frac{k}{2}\\ 0, & \;\text{otherwise}\; \end{array} \end{array}$$ (1)

Ensemble pruning, selective ensembles, ensemble selection and ensemble thinning are all different names given to the same task—reducing ensemble sizes. Pruned ensembles exhibit better performance and robustness with lower computational and memory costs [28], compared to traditional ensemble techniques [29, 30]. The three most popular ensemble pruning techniques are ranking, clustering and optimization [31], and this study focuses on the latter. Among the optimization techniques for ensemble pruning the most commonly used are evolutionary algorithms, semi-definite programming and hill climbing [32–34].

GASEN-b was one of the earliest algorithms for ensemble pruning, and was introduced by [32]. The ensemble is represented as a bit string, with each decision tree model using a bit. The bit string representation provides a direct mechanism of adding or removing classifiers, as opposed to a weighting mechanism with a predefined threshold. A similar approach was also used in [6] to select/remove classifiers from a heterogeneous pool of networks.

Zhang et al. [33] chose a quadratic integer programming approach for pruning. The weights were kept binary and the size of the final ensemble was prefixed. In terms of computational complexity, the algorithm could run in polynomial time.

Hill climbing methods generally use either forward selection or backward elimination of classifiers, and include various performance measures, e.g. diversity, weighted accuracy [35–39]. More recently, human-like foresight has been used as a measure to prune ensembles via hill climbing [34].

In this study, a pool of optimized EWNNs is pruned using genetic algorithms so that a better prediction model is obtained. The approach follows the GASEN-b mechanism [32] of pruning classifiers directly through bits so that to reduce the amount of parameter tuning. Our method introduces a fitness function that involves the sum of two accuracy measures: the accuracy of each individual classifier; and the ensemble accuracy using the voting method.

### Network performance measures

There are many performance measures for binary classification problems available in the literature. Power [40] investigated those measures and generalized them for multiclass problems. Next, we present the measures used in this work:

- Training Accuracy (*Tr*_*acc*_): fraction of correctly classified samples in the training set U.
- Test Accuracy (*Te*_*acc*_): fraction of correctly classified samples in the test set V. This is also known as the classification accuracy, and expressed as *Te*_*acc*_ = (*TP* + *TN*)/(*P* + *N*). TP represents true positive cases, i.e. accurate classification of control (non-diseased) samples; TN represents true negative cases, i.e. accurate classification of diseased samples; and (*P* + *N*) is the total number of positive and negative test samples.
- Sensitivity (*Sens*): measurement of the fraction of true positive cases, mathematically expressed as *Sens* = *TP*/(*TP* + *FN*). FN is the number of false negatives and reflects the more serious mistake of classifying a disease sample as control.
- Specificity (*Spec*): measurement of the fraction of true negative cases, mathematically represented as *Spec* = *TN*/(*TN* + *FP*). FP reflects the misclassification of control samples as diseased ones.
- Mathew’s Correlation Coefficient (*MCC*): is a balanced measure of quality for binary classification problems, normally used if classes are unbalanced. The measure was introduced in [41] and is expressed as:
  $MCC=W1/\sqrt{W2}\text{,}\;\text{where}$
  W1=TP·TN−FP·FN
  W2=(TP+FP)·(TP+FN)·(TN+FP)·(TN+FN)

## Experimental simulations

This section provides a description of the three biomedical datasets, references to some related studies and the experimental settings for the proposed approach. An overview of the datasets’ characteristics is given in Table 1.

**Table 1:** Datasets’ characteristics used in this study.

Datasets (References)	Abbrev.	Class distribution (control,diseased)	Number of Features
Digital Database for Screening Mammography [42]	DDSM	(100,100)	6
Little’s Parkinson’s Disease [43, 44]	LPD	(48,147)	22
Sakar’s Parkinson’s Disease [45]	SPD	(520,520)	26

### Datasets

#### Digital Database for Screening Mammography (DDSM)

The DDSM is an online repository of mammographic images (available at: http://marathon.csee.usf.edu/Mammography/Database.html) with different resolutions and obtained from various hospitals [46, 47]. The suspicious areas on the mammograms were manually marked by two experienced radiologists. For analysis, these markings are represented as chain codes and hence can be extracted easily. In the dataset used by [48], 200 mammographic images scanned by a HOWTEK scanner at 43.5 micron per pixel spatial resolution were downloaded and extracted via the chain code. That dataset had an equal number of benign and malignant samples. Even though [48] derived 25 features from the extracted region, only 6 of the features were actually investigated in the present study, in order to provide a fair comparison with previous works that used the same dataset [11, 49]. Among those 6 features, there are 4 BIRADS (Breast Imaging Reporting and Data System established by [50]) lexicon features: *mass shape, mass margin, assessment, breast density*, specified by an expert radiologist; and 2 features: *Patient age* and *subtlety*, that were extracted from the individual mammographic records.

#### Little’s Parkinson’s Dataset (LPD)

This dataset (available at: http://archive.ics.uci.edu/ml) was acquired from the online machine learning database repository from the University of California at Irvine (UCI) [51, 52]. It is a challenging, imbalanced dataset that has been investigated previously by several researchers [9, 53–55]. It contains 195 samples, each with 22 different biomedical voice measurements. These voice measurements were taken from 31 individuals, where 23 had Parkinson’s disease. Each patient has between 6 and 7 records in the data set, totalling 195 samples.

#### Sakar’s Parkinson’s Dataset (SPD)

The dataset by Sakar et al. [45] is a recent entry (from 2014) in the UCI database (available at: http://archive.ics.uci.edu/ml) [43]. The dataset contains multiple speech recordings that include sustained vowels (a, o, u), numbers from 1 to 10, four short rhyming sentences and nine turkish words from 40 individuals. These recordings sum up to 26 records per individual. Half of the individuals are diagnosed with Parkinson’s disease and the other half represents control subjects.

### Training and test sets

**Fig 3:**
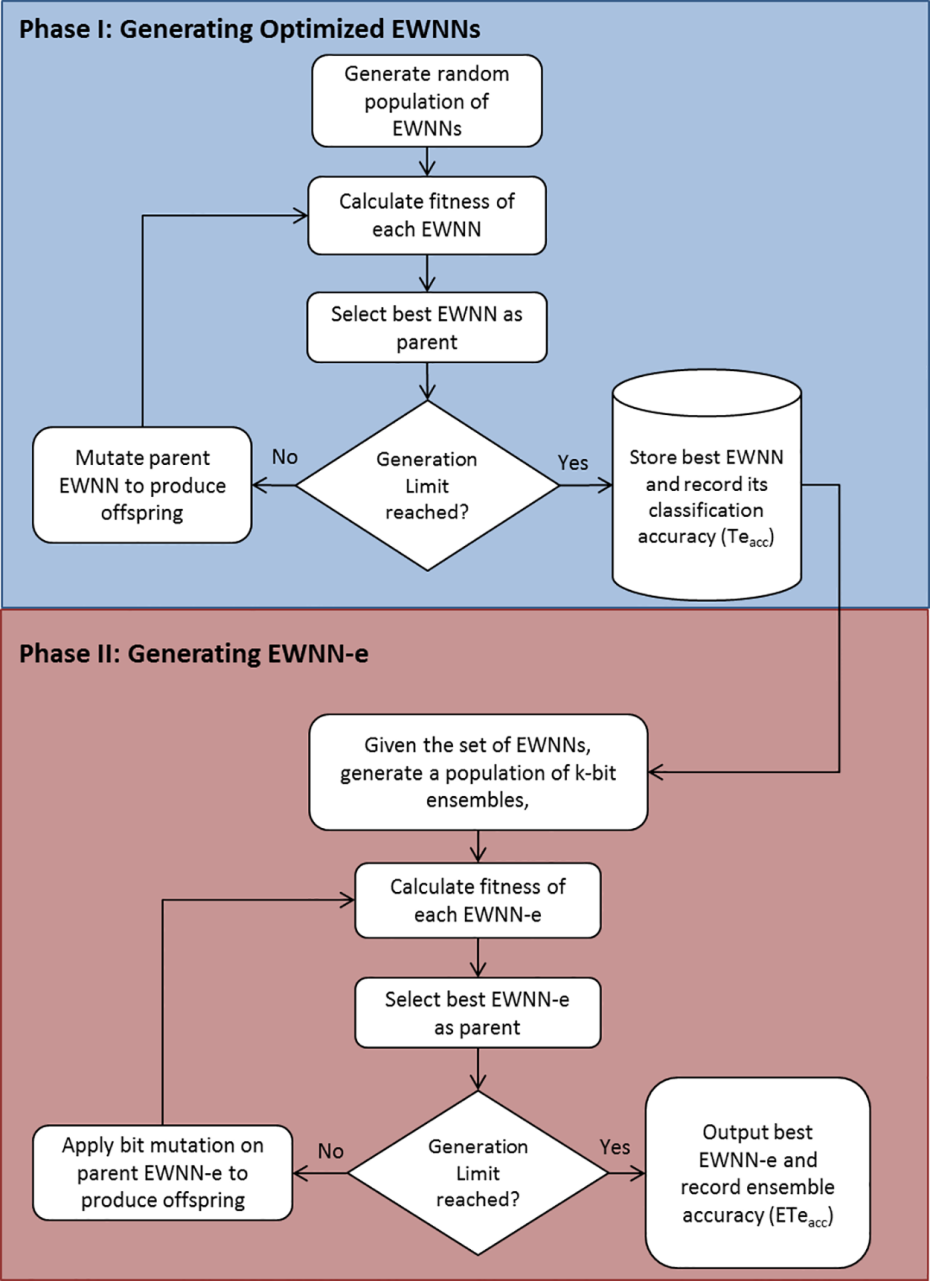
Flowchart of the two phases of the approach. Phase I is the process of generating optimized EWNNs. Phase II uses the optimized EWNNs to generate the ensemble of EWNNs.

For all the datasets, the data was divided into 90% training and 10% test data. The proposed approach is divided into two main phases as shown in Fig 3. Phase I creates optimal EWNNs from cross validation folds conducted on the 90% training and validation data and the average classification accuracy *Te*_*acc*_ for the EWNNs was reported. The optimal EWNNs were then used by next phase. Phase II uses genetic algorithm to prune the optimal EWNN classifiers where the separate test set was used and a final ensemble classification accuracy *ETe*_*acc*_ was then reported.

In both LPD and SPD datasets, individuals had different numbers of records. Thus, if more than half of the individual’s records are classified as Parkinson’s disease, then the individual itself is classified as Parkinson’s disease (diseased). This approach was adopted from [45, 54] in order to avoid over-fitting, as the frequency response of the records of the same patient are potentially very similar.

### Approach

#### Phase I: Generating optimized EWNNs

1. EWNN initialization: An EWNN genome requires the initialization of the number of wavelons, the different parameters of each wavelon, and the wavelet function(s).
  - The number of wavelons is critical as too many wavelons would likely result in over-fitting and too few would not capture the variability of the data [56]. The three datasets have been investigated in detail under different parameter settings and those are reported in [15]. The best configurations from that study were adopted here. The number of wavelons used for each dataset is shown in Table 2.
  - Selection of an appropriate activation function depends on the data itself, but the Mexican hat wavelet has performed satisfactorily in many applications [56]. For the DDSM, the present study uses a heterogeneous WNN with four possible activation functions. For the remaining case studies, we used a homogeneous WNN that uses the Mexican hat wavelet as activation function.
  - Each wavelon is represented by matrices of inputs *x*_*n*_ ∈ [1, *Feat*]; switches *c*_*n*_ ∈ {0, 1} where 0/1 indicates non-connected/connected features, respectively; input weights *w*_*x*_*nm*__ ∈ [−1, +1]; scale parameters *α*_*nm*_ ∈ [0, 1]; translation parameters *β*_*nm*_ ∈ [−∞, +∞]; rotation parameters *R*_*nm*_ ∈ [−1, +1]; as well as categorical values representing the type of wavelet function *ψ*_*m*_ ∈ [1, *number*_*waveletFunctions*_]; wavelon weights *wt*_*m*_ ∈ [−1, +1]; and active neurons *ot*_*m*_ ∈ {0, 1}, where 0/1 represents an inactive/active hidden neuron, respectively. The parameters of each wavelon are initialized uniformly at random, within the corresponding ranges of possible values.
2. Population Size: There are two basic types of evolutionary strategies: (*μ*, λ)-ES and (*μ* + λ)-ES [57]. *μ* represents the parent population and λ refers to the number of offspring produced in a generation. In (*μ*, λ)-ES, offspring replaces the parents as the *μ* fittest are selected from λ, while in (*μ* + λ)-ES, the *μ* fittest are selected from both parents and offspring for the next generation. The value of *μ* and λ used for the different case studies are shown in Table 2.
3. Fitness evaluation: All individuals in the population are evaluated and sorted based on their accuracies and mean square error where the best individual is promoted as parent to the next generation. The purpose of using two dimensional sorting is to promote networks with uncorrelated evaluation metrics in generations ahead.
4. Mutation: A 1% mutation rate is used to generate new EWNNs, similar to [15]. Mutation occurs in three different ways. For continuous parameters, such as input weights, wavelon weights, translation, rotation, dilation parameters and the bias, values are perturbed by adding a small percentage of the current value. For binary parameters, e.g. switch, the value is inverted from 0 to 1 or 1 to 0. For the third type of mutation, a network input is randomly changed to another input feature in the feature list, or similarly, a wavelet function is randomly changed to another wavelet function in the list.
5. Termination condition: The simulations stop at 2,000 generations. We observed that this value is sufficient for the evolutionary process to converge to a high-quality solution. The optimal EWNNs are later used in Phase II to create the ensembles. A total of 50 independent evolutionary runs were executed for each of the cross-validation folds.

**Table 2:** Main parameter settings of the evolutionary wavelet neural networks for the different datasets.

Parameters	DDSM	LPD	SPD
Wavelons	4	5	5
Input to wavelons	6	6	26
(*μ* + λ)-ES [57]	(1+25)	(1+25)	(3+20)

#### Phase II: Genetic algorithm-based ensemble

Given the set of optimized EWNN ensembles, the next step is to prune them. This stage uses another genetic algorithm as follows:

1. Chromosome *Chr* representation: A *k*-bit string is used to represent an ensemble with the optimized EWNNs. A bit value of 1 indicates that the classifier is actively used in the ensemble; 0 otherwise.
2. Population size: After a number of preliminary tests, we decided for an (*μ*+λ)-evolutionary strategy with *μ* = 3 parents and λ = 25 offspring in each generation. For ensemble pruning, having 3 parents considerably reduced the risk of premature convergence and at the same time kept the evolutionary process under a reasonable selective pressure.
3. Fitness evaluation: The fitness value of each chromosome is evaluated as in Eq 2. It is an average of the individual accuracies *Tr*_*acc*_ of the active EWNNs and their ensemble training accuracy *Ens(U)*, where the objective is to maximize the average accuracies.
  $$\begin{array}{c} FE\left(Chr\right)=0.5(Tr_{acc}+Ens\left(U\right)) \end{array}$$ (2)
4. Mutation: After pilot tests, mutation rate was set to 1% for all simulations, and the strategy used was bit-swap.
5. Termination condition: The limit for the number of generations was set at 1,000. Ensemble accuracy was found not to improve after few hundred generations.

The program starts with random chromosomes that are evaluated based on the fitness function in Eq 2. The best individuals are selected as parents and thus preserved for the next generation—all other individuals are removed. Then, λ offspring are produced by mutating the parents. Every offspring is evaluated and added to the next generation. The process continues until the number of generations limit is reached. The best parent’s ensemble accuracy *ETe*_*acc*_ on the test set is then reported.

## Results and discussion

Did the ensemble of EWNNs perform better? The performance of the evolutionary ensemble method is shown in Table 3. Classification accuracy *Te*_*acc*_, ensemble classification accuracy *ETe*_*acc*_, sensitivity *Sens*, specificity *Spec* and Mathew correlation coefficient *MCC* are reported for the three datasets. The ensemble approach improves the classification accuracy by up to 23.7 percentage points (*Te*_*acc*_ vs. *ETe*_*acc*_), compared to individual EWNN classifiers. For the DDSM dataset, the ensemble approach improved the performance of the network from 89.0% to 95.5%. An MCC score of 91.0% also indicates a very high classification accuracy. For the LPD dataset the accuracy increased from 92.9% to 100%, and for the SPD dataset it increased from 66.3% to 90.0%.

**Table 3:** Performance of the ensemble EWNN on the different case studies. Notice the increase in accuracy of the classifiers when an ensemble approach is adopted (second column).

Datasets	*Te*_*acc*_%	*ETe*_*acc*_%	Sens.%	Spec.%	MCC%
DDSM	89.0	95.5	95.0	96.0	91.0
LPD	92.9	100.0	100.0	-	100.0
SPD	66.3	90.0	93.0	97.0	87.0

**Fig 4:**
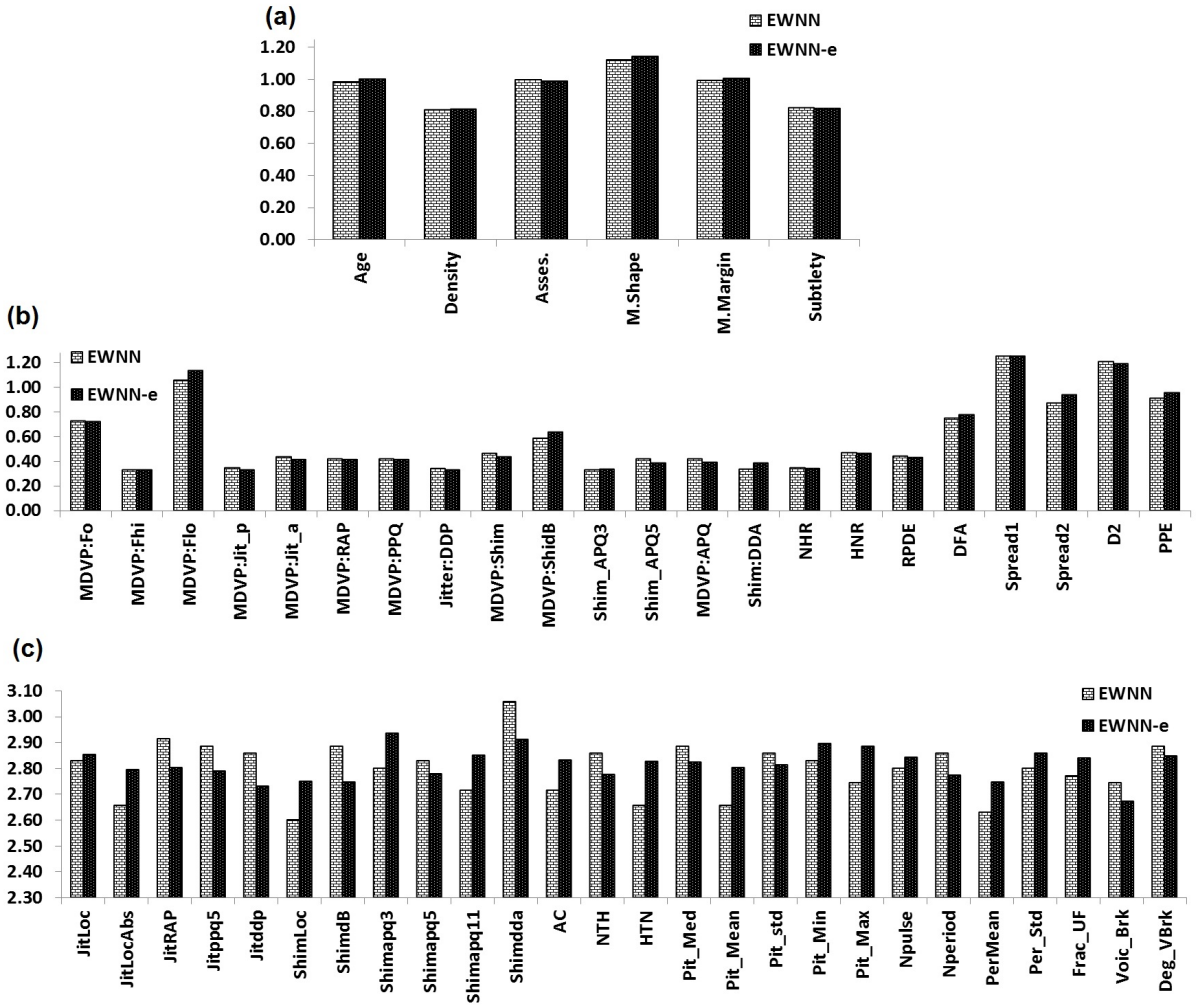
Identification of significant features. The figure shows the average number of connections per feature within the EWNN (over 50 independent evolutionary runs) and its ensemble EWNN-e (over the active classifiers), for the datasets: (a) DDSM [42], (b) LPD [44], and (c) SPD [45]. For all three datasets, and for all features, the average is higher than zero indicating that no feature should be completely removed from analysis. For illustration purposes, consider the example of feature Age in (a). The correct way to interpret the values is that the feature is connected to 1 wavelon on average, considering the 50 runs of EWNN. Details on the features can be found in the referenced papers [42, 45, 44].

What were the significant features identified by the process? Fig 4 is the averaged connected features for all datasets, across 50 independent runs in EWNN, and the number of active classifiers in the EWNN-e. In a standard WNN all features are connected to every wavelons in the hidden layer. While in EWNNs (from Fig 4), there is some variability in how often these features are connected. This indicates the flexibility of pruning features (during training) at the hidden layer, as opposed to the input layer, for which many feature reduction methods already exist. For the DDSM dataset, *mass margin*, *patient age*, *mass shape* and *assessment* were the top four features that had an impact on performance—similarly to [48]. For the LPD dataset, *spread1* and *D2* were the top two features—similarly to [53]. The trend of feature selection was found to be same for both the EWNN and EWNN-e networks for all datasets except SPD. For the SPD dataset, *Shimmer*_*apq*3_ is the top feature in the ensemble network, whereas *Shimmer*_*dda*_ is the top one for the individual EWNNs. This drift in frequency of feature selection indicates possible significance of the feature in the ensemble domain.

**Fig 5:**
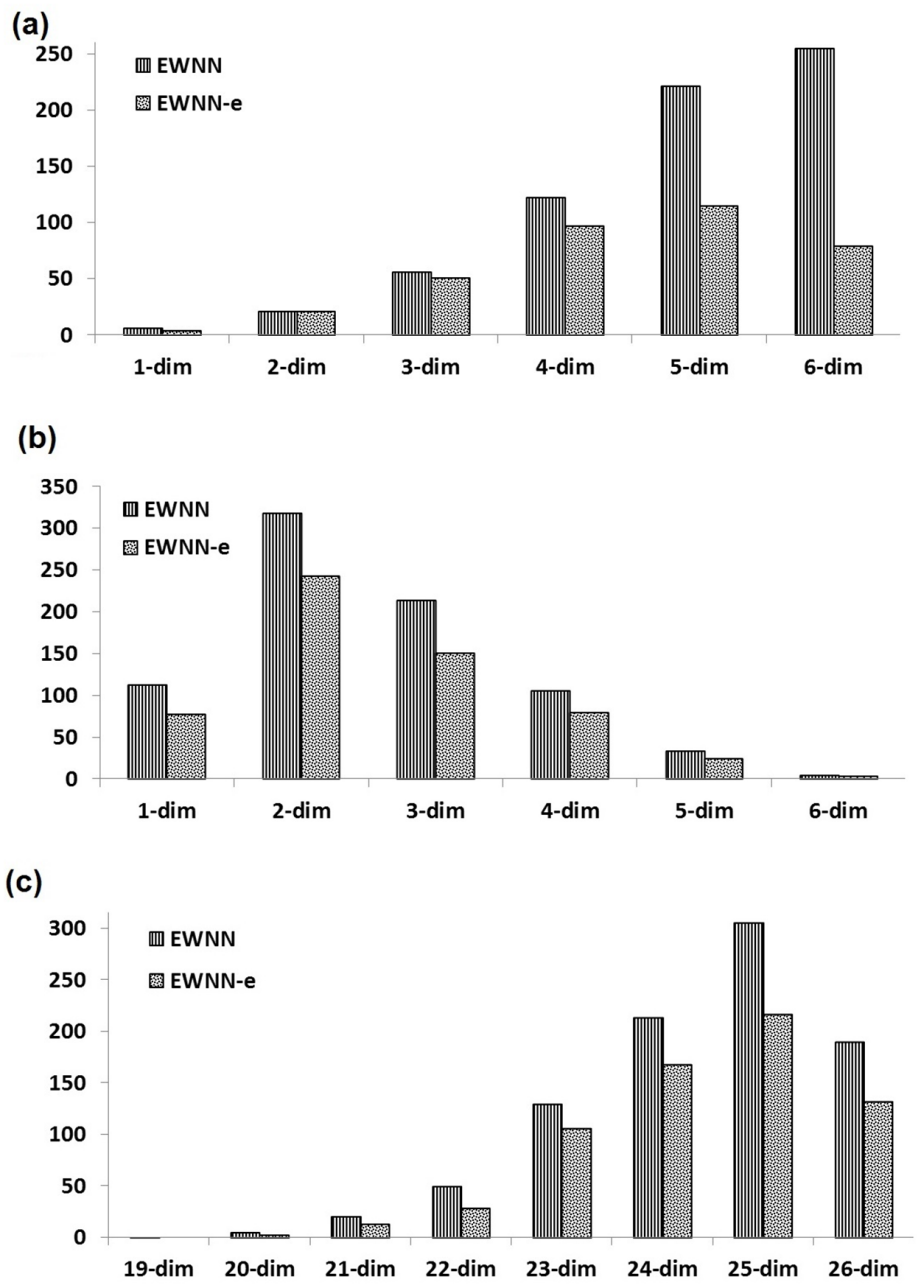
Should every wavelon be fully connected? Summation of wavelons’ dimensionality for individual EWNNs (over 50 independent evolutionary runs); and for the ensemble EWNN-e (over the number of active classifiers), for the three datasets (a) DDSM, (b) LPD and (c) SPD. Note the overall increasing trend for DDSM, with a larger number of high-dimensionality wavelons (except for EWNN-e which shows a decrease in the number of 6-dimensional wavelons). For LPD we see a concentration between 2- to 4-dimensional wavelons for both individual and ensemble EWNNs. Finally, for SPD, we see a concentration at the higher dimensions. These results indicate that having the features connected to all wavelons is not necessarily the most appropriated choice.

Should every wavelon be fully connected? The connectivity or dimensionality of a wavelon is determined by the number of active or connected inputs. Fig 5 displays the sum of the wavelons’ dimensions for each dataset, over 50 independent runs and, over the number of active classifiers in the final EWNN-e, across the 10 folds. The frequency of each wavelon dimension is lower in the ensemble network, as classifiers are pruned. The ensemble networks exhibited different trends, depending on the dataset. Interestingly, for the DDSM dataset we observed a reduction in the number of 6-dimensional wavelons, thus indicating that fully connected EWNNs were not part of the ensemble network. The frequent occurrence of wavelons with lower dimensions indicates that WNNs should be given the flexibility to adjust their input, in contrast to a standard WNNs, where all inputs are connected [16].

How many classifiers are necessary to create an effective ensemble? The average number of EWNNs in the ensemble networks for the datasets is shown in Table 4. The ensemble networks combine around 1/3 (14-17) of the 50 available EWNNs, and they improved both speed and performance, compared to the non-ensemble approach.

**Table 4:** Average number of active EWNNs in the ensembles, using 10-fold cross-validation, and across the three datasets. The average is calculated over 50 independent runs.

Datasets	Average EWNNs
DDSM	14.50
LPD	16.80
SPD	16.80

From Table 5, it can be concluded that the proposed method generated either competitive or better results in comparison to existing techniques. An advantage of EWNN-e is that it does not require pre-processing for feature pruning, which is present in some of the comparison methods. Given the results, it can be stated that the ensemble version of EWNN classifiers is a suitable approach for predictive analysis. Just for clarification purposes, and to put the results into context, for the DDSM dataset, the accuracy reported for NN-e was achieved with an ensemble of 127 classifiers [11], as opposed to the average of only 14.50 in the proposed method. That is, NN-e has a better performance for that dataset, but the classifier is much more complex than the classifiers obtained by our approach.

**Table 5:** Comparison between EWNN/EWNN-e and the different classifiers found in the literature for the DDSM, LPD and SPD datasets. In the case of LPD, EWNN-e outperformed all methods reported in literature, reaching a test accuracy of 100%.

**DDSM**	
**Algorithm**	**Test accuracy (%)**
SCBDL [58]	97.50
SCNN [59]	94.00
MCSVM [60]	94.50
NN-e [11]	99.00
LCA-e [10]	94.00
**EWNN-e**	95.50
**LPD**	
**Algorithm**	**Test accuracy (%)**
SVM+RBF-e [9]	88.71
SVM+LADTree-e [9]	92.82
SVM+J48-e [9]	92.3
SVM+KSTAR-e [9]	96.41
SVM+IBk-e [9]	96.93
**EWNN-e**	100.00
**SPD**	
**Algorithm**	**Test accuracy (%)**
**EWNN**	66.3
**EWNN-e**	90.00

## Conclusion

Ensemble approaches aim at combining the classification power of individual classifiers ultimately improving the overall performance of the system. The current study contributes to the literature of ensemble classifiers by proposing an ensemble of evolutionary wavelet neural networks (EWNN-e).

The performance of the EWNN-e has been validated on three biomedical datasets. The pruned EWNN-e used less than 1/3 of the available EWNNs and resulting in better performance. For one of the datasets, the method achieved a testing accuracy of 100%, whereas the best approach reported in literature to date had reached 96.9% only.

Each EWNN used all features available, but features were not connected to every wavelon in the network. In other words, the proposed method prunes features at the hidden layer level, instead of at the input layer level.

The dimensionality of the wavelons is represented by the number of active inputs. The trend of the average sum of wavelons’ dimensionality in the Parkinson’s disease datasets was same for both EWNNs and EWNN-e. While for the Breast Cancer dataset (DDSM) the wavelons’ dimensionality of a fully connected wavelon were reduced in the EWNN-e. This indicates that WNNs should be provided with the flexibility to adjust their network inputs, as opposed to a conventional WNNs, where all inputs are forced to be connected.
